# Phase I/II sequencing study of azacitidine, epacadostat, and pembrolizumab in advanced solid tumors

**DOI:** 10.1038/s41416-023-02267-1

**Published:** 2023-04-22

**Authors:** Jason J. Luke, Marwan Fakih, Charles Schneider, E. Gabriela Chiorean, Johanna Bendell, Rebecca Kristeleit, Razelle Kurzrock, Sarah P. Blagden, Irene Brana, Laura W. Goff, Kevin O’Hayer, Ryan Geschwindt, Michael Smith, Feng Zhou, Aung Naing

**Affiliations:** 1 UPMC Hillman Cancer Center, Pittsburgh, PA USA; 2 City of Hope Comprehensive Cancer Center, Duarte, CA USA; 3 Abramson Cancer Center, University of Pennsylvania, Philadelphia, PA USA; 4 University of Washington School of Medicine, Fred Hutchinson Cancer Research Center, Seattle, WA USA; 5 Sarah Cannon Research Institute/Tennessee Oncology, Nashville, TN USA; 6 Guy’s and St. Thomas’ NHS Foundation Trust, London, UK; 7 University of California San Diego School of Medicine, La Jolla, CA USA; 8 Early Phase Clinical Trials Unit, University of Oxford, Oxford, England UK; 9 Vall d’Hebron University Hospital, Vall d’Hebron Institute of Oncology (VHIO), Barcelona, Spain; 10 Vanderbilt-Ingram Cancer Center, Vanderbilt University Medical Center, Nashville, TN USA; 11 Incyte Corporation, Wilmington, DE USA; 12 The University of Texas MD Anderson Cancer Center, Houston, TX USA

**Keywords:** Cancer immunotherapy, Cancer epigenetics

## Abstract

**Background:**

Indoleamine 2,3-dioxygenase 1 (IDO1), an interferon-inducible enzyme, contributes to tumor immune intolerance. Immune checkpoint inhibition may increase interferon levels; combining IDO1 inhibition with immune checkpoint blockade represents an attractive strategy. Epigenetic agents trigger interferon responses and may serve as an immunotherapy priming method. We evaluated whether epigenetic therapy plus IDO1 inhibition and immune checkpoint blockade confers clinical benefit to patients with advanced solid tumors.

**Methods:**

ECHO-206 was a Phase I/II study where treatment-experienced patients with advanced solid tumors (*N* = 70) received azacitidine plus an immunotherapy doublet (epacadostat [IDO1 inhibitor] and pembrolizumab). Sequencing of treatment was also assessed. Primary endpoints were safety/tolerability (Phase I), maximum tolerated dose (MTD) or pharmacologically active dose (PAD; Phase I), and investigator-assessed objective response rate (ORR; Phase II).

**Results:**

In Phase I, no dose-limiting toxicities were reported, the MTD was not reached; a PAD was not determined. ORR was 5.7%, with four partial responses. The most common treatment-related adverse events (AEs) were fatigue (42.9%) and nausea (42.9%). Twelve (17.1%) patients experienced ≥1 fatal AE, one of which (asthenia) was treatment-related.

**Conclusions:**

Although the azacitidine-epacadostat-pembrolizumab regimen was well tolerated, it was not associated with substantial clinical response in patients with advanced solid tumors previously exposed to immunotherapy.

## Introduction

Tumor cells can evade host immune responses by exploiting immune checkpoint pathways [1]. These pathways, including programmed death receptor-1 (PD-1) and cytotoxic T lymphocyte antigen-4 (CTLA-4), downregulate effector T cells to protect healthy tissue from immunologic damage [1, 2]. Treatments that counter tumor-mediated suppression of host immunity can help overcome cancer resistance. For example, the PD-1 inhibitor pembrolizumab has been shown to confer durable antitumor responses in multiple types of solid tumors [3]. However, 40–60% of patients do not respond to immune checkpoint inhibition [4].

The mechanisms responsible for resistance to immune checkpoint inhibition are not fully defined, but treatment responsiveness has been associated with the presence of an inflamed tumor microenvironment [5], which is characterized by infiltrating T cells [6] and the expression of interferon (IFN)-associated genes [7, 8]. However, T-cell-inflamed tumors can also be immunosuppressive, as illustrated by the presence of FoxP3^+^ regulatory T cells [9] and overactivation of the tryptophan–kynurenine–aryl hydrocarbon receptor (Trp–Kyn–AhR) pathway [10]. As multiple immune inhibitory mechanisms act concurrently within the tumor microenvironment [1], combination treatment may be required to optimize patient outcomes.

Immunotherapy strategies targeting indoleamine 2,3-dioxygenase 1 (IDO1), a tryptophan-catabolizing enzyme, may enhance the efficacy of immune checkpoint blockade. IDO1 contributes to tumor immune tolerance by inhibiting T-cell proliferation, inducing T-cell apoptosis, promoting the differentiation of naïve T cells into regulatory T cells, and regulating the pool of peptides available for antigen presentation [11–14]. Elevated levels of IDO1 correlate with reduced survival in patients with cancer [15–17]. Co-expression of IDO1 and programmed death-ligand 1 (PD-L1; the ligand of PD-1) has been observed in multiple cancer types [9, 18–20]. Because IDO1 is IFN-inducible, the activation of IFN-secreting T cells following treatment with an immune checkpoint inhibitor may increase IDO1 levels, leading to a dampened immune response [21]. Therefore, regimens that combine IDO1 inhibition with immune checkpoint blockade represent an attractive therapeutic approach for patients with T-cell–inflamed tumors.

In preclinical studies, inhibition of both IDO1 and an immune checkpoint pathway provided greater control of tumor growth than immune checkpoint inhibition alone [22, 23]. These observations provided the basis for clinical evaluation of epacadostat, a potent and highly selective oral inhibitor of IDO1 [24], in combination with immune checkpoint blockade for the treatment of patients with solid tumors. Across several Phase I/II studies, treatment with epacadostat plus immune checkpoint blockade (pembrolizumab, ipilimumab, or nivolumab) demonstrated activity in these patients [25–28]. However, in a randomized Phase III trial of epacadostat 100 mg twice daily (BID) plus pembrolizumab versus placebo plus pembrolizumab in patients with advanced melanoma, treatment with epacadostat plus pembrolizumab did not result in improved progression-free survival (PFS) or overall survival (OS) relative to treatment with placebo plus pembrolizumab [29]. Because of these findings, ongoing, randomized Phase II and Phase III studies of epacadostat and PD-1 inhibition in other solid tumor types were prematurely terminated.

Epigenetics refers to alterations in gene expression that occur independently of changes to inherited gene sequences. Epigenetic changes in malignant cells can lead to the upregulation of genes that promote cancer progression [30]. Epigenetic modulators—such as DNA methyltransferase (DNMT) inhibitors (eg, azacitidine), histone deacetylase inhibitors, bromodomain and extra-terminal protein (BET) inhibitors, and lysine-specific demethylase 1 inhibitors—are hypothesized to have the ability to convert tumors from a non-inflamed (“cold”) state to an inflamed (“hot”) state and to sensitize tumors to treatment with an immune checkpoint inhibitor [31–33]. For example, in non-small cell lung cancer (NSCLC) and other epithelial cancer cell lines, treatment with azacitidine led to the upregulation of PD-L1, as well as genes and pathways involved in innate immunity, adaptive immunity, and immune evasion [32]. Specifically, azacitidine-mediated inhibition of DNA methylation enhances immune signaling through a viral defense pathway that triggers an interferon response [34].

Clinical data on the antitumor activity of combination treatment with an epigenetic modulator and an immune checkpoint inhibitor were limited at the time when the current study was designed. However, in a prior study, five patients with NSCLC previously treated with azacitidine and the histone deacetylase inhibitor entinostat exhibited durable (>6 months) disease control [32]. Collectively, the available data suggested that epigenetic agents may prime the tumor microenvironment for immune checkpoint inhibition by triggering an IFN response, and that concurrent blockade of IFN-regulated resistance pathways via IDO1 could provide further clinical benefit. To this end, the Phase I/II ECHO-206 study assessed the safety and efficacy of epigenetic priming when used in combination with epacadostat and pembrolizumab in patients with advanced solid tumors.

## Methods

### Study design and participants

ECHO-206 (NCT02959437) was an international, open-label, Phase I/II study in which patients received an epigenetic priming regimen and an immunotherapy doublet consisting of epacadostat and pembrolizumab. The study was undertaken at 11 centers in the United States, United Kingdom, and Spain. Patients enrolled in Phase I had confirmed advanced or metastatic solid tumors and had failed prior standard treatment (no limit to the number of prior regimens). Phase II planned to enroll several refractory solid tumors and ultimately included NSCLC and microsatellite stable (MSS) colorectal cancer (CRC) cohorts. NSCLC patients with prior anti-PD(L)1 were selected because this tumor type is known to be responsive to anti-PD(L)1 therapy, and combination with an epigenetic modifier was hypothesized to improve or restore antitumor activity. MSS CRC does not have a high mutational burden, and the lack of immunogenicity makes this histology unlikely to respond to immunotherapy alone. MSS CRC became a tumor type of interest based on preclinical data suggesting that epigenetic reprogramming could increase the immunogenicity of the tumor and synergize with anti-PD(L1) immunotherapy to promote antitumor activity [35, 36].

Patients in both study phases were adults (≥18 years) with disease that was measurable per Response Evaluation Criteria in Solid Tumors (RECIST) v1.1 [37], had an Eastern Cooperative Oncology Group (ECOG) performance status score of 0 or 1, and were willing to undergo pre-treatment and on-treatment tumor biopsies. Exclusion criteria included the presence of abnormal laboratory values, such as absolute neutrophil count <1.5 × 10^9^/L; platelet count <100 × 10^9^/L; hemoglobin <8 g/dL; serum creatinine ≥1.5 times the upper limit of normal (ULN); aspartate aminotransferase, alanine aminotransferase, or alkaline phosphatase ≥2.5 times ULN; and serum albumin <3 g/dL. Other exclusion criteria included lack of recovery to grade ≤1 from the toxic effects of prior therapy; presence of active or inactive autoimmune disease or syndrome; known active central nervous system metastases and/or carcinomatous meningitis; presence of active infection requiring systemic therapy; history of grade 3–4 immune-related treatment-emergent adverse events (TEAEs; part I dose-escalation only); and use of chemotherapy, a PD-1 pathway-targeted agent, or immunosuppression (for any reason) within 14 days of the first dose of study drug.

The Phase I study, which sought to determine the maximum tolerated dose (MTD) or pharmacologically active dose (PAD) of the triple-drug combination, employed a 3 + 3 + 3 study design. The Phase II study employed a Simon two-stage design and consisted of dose-expansion cohorts (to assess the safety and efficacy of the MTD or PAD) and treatment-sequencing, tumor-biopsy cohorts (to evaluate epigenetic changes, as well as changes in the tumor microenvironment). Patients in the dose-expansion cohorts had either NSCLC or MSS CRC and were required to undergo two biopsies, one before treatment and one prior to day 1 of cycle 3 (see Additional file 1: Supplementary Fig. S1). Patients in the NSCLC cohort must have progressed on a prior PD-(L)1 inhibitor. Under the Simon two-stage design, eight patients with NSCLC and eight with MSS were enrolled in Stage 1. If no response was observed in a cohort (NSCLC or MSS CRC), the cohort(s) were discontinued. If ≥1 response was observed in a cohort, 19 additional patients were enrolled in that cohort under Stage 2. Patients in the treatment sequencing, tumor-biopsy cohorts had MSS CRC, head and neck squamous cell carcinoma (HNSCC), melanoma, or urothelial carcinoma and were required to undergo three biopsies per the schedule depicted in Supplementary Fig. 1. With the exception of MSS CRC, these tumor types were selected because they have historically demonstrated responses to immunotherapy with checkpoint inhibitors, and we hypothesized that epigenetic modification could restore antitumor activity. Preliminary response data for combination pembrolizumab and epacadostat in urothelial cancer [38] and HNSCC [39] also suggested promising activity at the time of the study. The following treatment sequences were examined (see Additional file 1: Supplementary Fig. S2): concurrent administration of azacitidine, epacadostat, and pembrolizumab (Groups A-1 and A-2); 7-day run-in with azacitidine followed by the addition of epacadostat and pembrolizumab (Group A-3); and epacadostat and pembrolizumab for one cycle followed by the addition of azacitidine (Group A-4).

### Sample size considerations

Part 1 dose escalation used a 3 + 3 + 3 design, and the sample size was determined by the frequency of dose-limiting toxicities (DLTs) and final number of dose levels tested before the MTD or PAD was established.

For Part 2 expansion, the sample size was based on the Simon 2-stage design. Based on a one-sided type I error of 0.05 and 80% power, a total of 27 patients with 8 subjects in Stage 1 would be required to demonstrate the desired response rate of 20% assuming the response rate for the historical control was 3%.

### Treatment

This study was originally designed to explore several epigenetic priming regimens, but azacitidine was the one tested due to the early study termination (discussed in the study conduct section).

Treatment cycles were 21 days long. Dose escalation began with azacitidine 75 mg, pembrolizumab 200 mg IV Q3W, and oral epacadostat 100 mg BID. Five doses of azacitidine (75 or 100 mg per dose) were administered by intravenous infusion or subcutaneous injection over days 1–7 in cycles 1 and 2. Only the subcutaneous formulation of azacitidine was available to patients in the European Union. Dose reductions of azacitidine were permitted for the management of TEAEs, with a maximum of two dose reductions regardless of the initial starting dose. Epacadostat (100 or 300 mg) was administered orally BID without regard to food. Dose reductions of epacadostat were permitted for the management of TEAEs, with a maximum of two dose reductions regardless of the initial starting dose. Pembrolizumab 200 mg was administered as a 30-min, intravenous infusion every 3 weeks beginning on day 1 of cycle 1. Dose reductions of pembrolizumab were not permitted, but doses could be delayed to manage TEAEs.

### Study conduct

The study was initiated on February 27, 2017, and a strategic decision was made on April 11, 2018, to permanently stop enrollment. This decision was based on the results of the Phase III ECHO-301/ KEYNOTE-252 study, which compared epacadostat plus pembrolizumab with placebo plus pembrolizumab in patients with advanced melanoma [29]. During the second interim analysis of KEYNOTE-252/ECHO-301, the external data monitoring committee concluded that PFS was not improved with combination therapy relative to pembrolizumab monotherapy and anticipated that OS would not reach statistical significance. Although there were no new safety concerns with epacadostat plus pembrolizumab compared with pembrolizumab monotherapy, the sponsors determined that additional exposure to epacadostat 100 mg BID would not yield clinically meaningful improvements.

ECHO-206 was conducted in accordance with Good Clinical Practice guidelines, the provisions of the Declaration of Helsinki, and applicable national and local regulatory requirements. The study protocol was approved by the independent ethics committee/institutional review board at each participating site, and all patients provided written informed consent.

### Endpoints

In Phase I, the primary endpoints were safety/tolerability and identification of the MTD or PAD. The MTD was defined as the highest dose at which less than one-third of patients (out of a minimum of six patients) experienced a DLT (see Additional file 2: Supplementary Table S1). Investigator-assessed ORR per RECIST v1.1 at the MTD or PAD was a secondary endpoint. ORR was defined as the percentage of patients with complete response (CR) or partial response (PR).

In Phase II, investigator-assessed ORR per RECIST v1.1 was the primary endpoint, and safety/tolerability was a secondary endpoint. Tumor assessments were performed every 9 weeks or more frequently if clinically indicated. Either computed tomography or magnetic resonance imaging could have been used, but investigators were instructed to use the same imaging technique throughout the study. After 12 months of study treatment, tumor assessments could have been performed every 12 weeks. Imaging was performed until documented disease progression, the start of new anticancer treatment, withdrawal of consent, death, or the end of the study. Safety/tolerability was evaluated throughout the study. TEAEs were coded per Medical Dictionary for Regulatory Activities v19.1 and graded per Common Terminology Criteria for Adverse Events (CTCAE) v4.03.

Changes in T-cell infiltration in the tumor microenvironment was a secondary endpoint in both Phase I and Phase II. To determine expression of PD-L1 in the tumor microenvironment, chromogenic immunohistochemistry was performed at Indivumed (Hamburg, Germany) using the 22C3 pharmDx assay (Agilent/Dako; Santa Clara, California, USA). Membranous anti-PD-L1 staining was semi-quantitatively evaluated using the H-score and tumor proportion score (TPS) per the manufacturer’s instructions. Changes in T-cell infiltration were assessed using 5-color, multiplex immunohistochemistry and analyzed by Indivumed. To quantify T cells, tissue sections were stained with antibodies specific to CD3 (2GV6; Roche/Ventana, Cat # 05278422001), CD8 (SP16; DCS, Cat # CI008C002), and FoxP3 (SP97; Spring Biotech, Cat # M3970; LS Bio, Cat # LS-C210349; Thermo Fisher, Cat # MA5-16365). To identify tumor regions, sections were stained with antibodies specific to pan-cytokeratin (polyclonal; Agilent/Dako, Cat # Z0622) or PMEL (HMB45; Leica, Cat # NCL-L-HMB45). Nuclei were stained with 4′,6-diamidino-2-phenylindole. TILs positive for CD3, CD8, and FoxP3 were quantified by counting the number of cells present within the tumor, as assessed by co-localization with pan-cytokeratin. Cell densities (cells/mm^2^) were quantified separately in tumor and stromal regions and as a composite of the total tissue section by OracleBio (North Lanarkshire, Scotland, UK) using HALO AI digital pathology software.

### Statistics

The response-evaluable population, which was used for the efficacy analysis, comprised patients who received ≥1 dose of any study drug and completed a baseline scan. Patients in the response-evaluable population also had to have ≥1 post-baseline scan, been on study for ≥70 days, or discontinued study treatment. The safety population comprised patients who received ≥1 dose of any study drug. Efficacy and safety data collected during Phase I and Phase II were pooled and summarized using descriptive statistics (SAS v9.4 or later). No evaluation of the effect of treatment sequence was conducted. Changes in T-cell infiltration in pre- and on-treatment biopsies was compared using the Wilcoxon matched-pairs signed rank test in GraphPad Prism v7.02. The cutoff date for these analyses was February 15, 2019.

## Results

### Patients

A total of 70 patients were enrolled, with two (2.9%) still on treatment at the time of data cutoff and seven (10%) ongoing as part of the safety follow-up. Sixteen patients derived from the dose-escalation cohort. Of the 54 patients from the dose-expansion cohort, six were assigned to Group A-1, nine to Group A-2, 23 to Group A-3, and 16 to Group A-4. Sixty-eight (97.1%) patients discontinued treatment, most commonly because of disease progression (80.0% [*n* = 56]).

The mean (standard deviation [SD]) age of all study participants was 56.5 (11.84) years. Most patients were male (67.1%) and white (87.1%), and the most common solid tumor type was MSS CRC (47.1%). Two patients with advanced, non-metastatic, disease at baseline were enrolled, and included 1 with NSCLC and another with cholangiocarcinoma. Approximately half (48.6%) of all patients had been previously treated with PD-(L)1–targeted therapy (see Table 1).

**Table 1:** Baseline demographics and disease characteristics.

	Epacadostat 100 mg (*n* = 62)	Epacadostat 300 mg (*n* = 8)	Total (*N* = 70)
Male, *n* (%)	43 (69.4)	4 (50.0)	47 (67.1)
Mean age, years (SD)	57.0 (11.92)	53.0 (11.31)	56.5 (11.84)
Age ≥65 years, *n* (%)	17 (27.4)	2 (25.0)	19 (27.1)
Race, *n* (%)			
White	56 (90.3)	5 (62.5)	61 (87.1)
Black/African American	3 (4.8)	0	3 (4.3)
Asian	2 (3.2)	0	2 (2.9)
Other	1 (1.6)	3 (37.5)	4 (5.7)
Solid tumor type, *n* (%)			
Colorectal	30 (48.4)	3 (37.5)	33 (47.1)
Gastric	1 (1.6)	0	1 (1.4)
HNSCC	8 (12.9)	0	8 (11.4)
Melanoma	13 (21.0)	0	13 (18.6)
NSCLC	6 (9.7)	0	6 (8.6)
Urothelial	4 (6.5)	0	4 (5.7)
Other	0	5 (62.5)	5 (7.1)
Disease status, *n* (%)			
Advanced	1 (1.6)	1 (12.5)	2 (2.9)
Metastatic	61 (98.4)	7 (87.5)	68 (97.1)
Prior lines of therapy, *n* (%)			
0	1 (1.6)	1 (12.5)	2 (2.9)
1	15 (24.2)	4 (50.0)	19 (27.1)
2	14 (22.6)	2 (25.0)	16 (22.9)
≥3	32 (51.6)	1 (12.5)	33 (47.1)
Prior treatment, *n* (%)			
Systemic therapy	62 (100.0)	8 (100.0)	70 (100.0)
Radiotherapy	28 (45.2)	4 (50.0)	32 (45.7)
Surgery	50 (80.6)	5 (62.5)	55 (78.6)
PD-1 and PD-L1–targeted therapy	32 (51.6)	2 (25.0)	34 (48.6)

*HNSCC* head and neck squamous cell carcinoma, *NSCLC* non-small cell lung cancer, *PD-1* programmed death receptor 1, *PD-L1* programmed death-ligand 1, *SD* standard deviation.

Of the 70 study participants, 62 (88.6%) received epacadostat at a dose of 100 mg, and eight (11.4%) received epacadostat at a dose of 300 mg. The mean (SD) duration of exposure to azacitidine, epacadostat, and pembrolizumab was 45.4 (34.47), 87.5 (81.94), and 72.7 (76.48) days, respectively.

No DLTs were reported during the Phase I portion of the study. The MTD was not reached, and a PAD was not determined. For this reason, the higher dose of azacitidine (100 mg) was chosen to explore in the expansion phase. Based on results from other ongoing trials of epacadostat combined with PD-(L)1 inhibition, epacadostat 100 mg BID showed a favorable tolerability profile and preliminary antitumor activity, and this dose was chosen for Phase III combination studies in several tumor types. This was also the rationale for choosing epacadostat 100 mg BID for the expansion cohorts in the current study.

### Response rates

All 70 patients recruited to Phase 1 and Phase 2 of ECHO-206 were included in the response-evaluable population. There were no CRs and four (5.7%) PRs; hence, the ORR was 5.7% (Table 2). PRs were observed in patients with the following tumor types: mesothelioma (dose-escalation cohort 3), urothelial carcinoma (Group A-3), melanoma (Group A-3), and MSS CRC (Group A-4). Thirteen patients had stable disease (SD) of at least 9 weeks duration, corresponding to a SD rate of 18.6%. The best response in most (62.9%) patients was progressive disease.

**Table 2:** Investigator-assessed best response per RECIST v1.1.

	Epacadostat 100 mg (*n* = 62)	Epacadostat 300 mg (*n* = 8)	Total (*N* = 70)
Best overall response, *n* (%)			
Complete response	0	0	0
Partial response^a^	3 (4.8)	1 (12.5)	4 (5.7)
Stable disease^b^	10 (16.1)	3 (37.5)	13 (18.6)
Disease control rate (CR + PR + SD)	13 (21.0)	4 (50.0)	17 (24.3)
Progressive disease	40 (64.5)	4 (50.0)	44 (62.9)
Not evaluable^c^	2 (3.2)	0	2 (2.9)
Patients who discontinued before the first tumor assessment	7 (11.3)	0	7 (10.0)

*MSS CRC* microsatellite stable colorectal cancer, *RECIST* Response Evaluation Criteria in Solid Tumors.

All patients received pembrolizumab 200 mg every 3 weeks and azacitidine 75 or 100 mg.

^a^Observed in patients with the following tumor types: mesothelioma (dose-escalation cohort 3), urothelial carcinoma (Group A-3), melanoma (Group A-3), and MSS CRC (Group A-4).

^b^Required measurements that met the stable disease criteria at least after the date of first dose at a minimum of 56 days (9 weeks—7-day window).

^c^Did not receive on-treatment scans due to disease progression.

Forty-nine (70.0%) study participants had pre-treatment biopsies that were evaluable (defined as tumor content ≥10%) for PD-L1 expression. The majority (53.1%) of samples were PD-L1–negative. No association between PD-L1 expression and treatment response was found. Because of the lack of clinical responses, no additional translational studies were performed.

### Safety and tolerability

All 70 study participants had ≥1 TEAE, with nausea (54.3%), fatigue (51.4%), vomiting (34.3%), and constipation (27.1%; Table 3) being most frequently reported (≥25%). In total, 85.7% of patients experienced an event that was considered treatment-related, with fatigue (42.9%) and nausea (42.9%) being the most frequently reported (≥25%). Thirty-seven (52.9%) patients had a grade 3–4 TEAE. Abdominal pain (8.6%), disease progression (5.7%), and nausea (5.7%) were the grade 3–4 TEAEs reported in >5.0% of patients. Serious TEAEs occurred in 44.3% of patients, with the following events reported in >2 patients: disease progression (*n* = 8), nausea (*n* = 5), abdominal pain (*n* = 3), back pain (*n* = 3), small intestinal obstruction (*n* = 3), and vomiting (*n* = 3). Twelve (17.1%) patients experienced a total of 14 TEAEs with a fatal outcome: disease progression (*n* = 9), asthenia (*n* = 1), brain injury (*n* = 1), brain edema (*n* = 1), lymphangitis carcinomatosis (*n* = 1), and respiratory failure (*n* = 1). Asthenia was the only TEAE leading to death that was considered by the treating physician to be treatment-related, specifically to epacadostat and pembrolizumab. The event of asthenia was accompanied by dizziness and loss of consciousness that resulted in a fall with head injury and confusional state. The patient was hospitalized, and magnetic resonance imaging with and without contrast revealed no areas of restricted diffusion suggestive of acute or subacute infarct, left mastoid effusion/mastoiditis, and mild mucosal thickening in bilateral mastoid air cells. The patient discontinued study treatment due to disease progression and entered hospice care. At hospital discharge, the final diagnosis was malignant neoplasm of the ascending colon, failure to thrive, and bilateral mastoiditis. Approximately 2 weeks after entering hospice care, the patient died.

**Table 3:** Safety summary.

Patients, *n* (%)	Epacadostat 100 mg (*n* = 62)	Epacadostat 300 mg (*n* = 8)	Total (*N* = 70)
Any TEAE^a^	62 (100.0)	8 (100.0)	70 (100.0)
Nausea	35 (56.5)	3 (37.5)	38 (54.3)
Fatigue	30 (48.4)	6 (75.0)	36 (51.4)
Vomiting	22 (35.5)	2 (25.0)	24 (34.3)
Constipation	16 (25.8)	3 (37.5)	19 (27.1)
Decreased appetite	12 (19.4)	5 (62.5)	17 (24.3)
Abdominal pain	13 (21.0)	2 (25.0)	15 (21.4)
Anemia	13 (21.0)	2 (25.0)	15 (21.4)
Diarrhea	12 (19.4)	3 (37.5)	15 (21.4)
Treatment-related TEAE^b^	52 (83.9)	8 (100.0)	60 (85.7)
Fatigue	24 (38.7)	6 (75.0)	30 (42.9)
Nausea	27 (43.5)	3 (37.5)	30 (42.9)
Vomiting	15 (24.2)	2 (25.0)	17 (24.3)
Injection site reaction	13 (21.0)	0	13 (18.6)
Decreased appetite	8 (12.9)	2 (25.0)	10 (14.3)
Constipation	5 (8.1)	2 (25.0)	7 (10.0)
Rash	5 (8.1)	2 (25.0)	7 (10.0)
Arthralgia	5 (8.1)	1 (12.5)	6 (8.6)
Diarrhea	5 (8.1)	1 (12.5)	6 (8.6)
Pruritus	6 (9.7)	0	6 (8.6)
Injection site erythema	5 (8.1)	0	5 (7.1)
Dizziness	3 (4.8)	1 (12.5)	4 (5.7)
Injection site pain	4 (6.5)	0	4 (5.7)
Any Grade 3–4 TEAE	31 (50.0)	6 (75.0)	37 (52.9)
Any serious TEAE	28 (45.2)	3 (37.5)	31 (44.3)
Any TEAE leading to dose reduction of any study drug	1 (1.6)^c^	0	1 (1.4)^c^
Any TEAE leading to interruption of any study drug	20 (32.3)	4 (50.0)	24 (34.3)
Any TEAE leading to discontinuation of any study drug	6 (9.7)	0	6 (8.6)
Any fatal TEAE	11 (17.7)	1 (12.5)	12 (17.1)

*TEAE* treatment-emergent adverse event.

All patients received pembrolizumab 200 mg every 3 weeks and azacitidine 75 or 100 mg.

^a^TEAEs (any grade) reported in ≥20% of patients in the total study population are presented.

^b^Treatment-related TEAEs (any grade) reported in ≥5% of patients in the total study population are presented.

^c^The doses of both azacitidine and epacadostat were reduced.

One (1.4%) patient experienced four TEAEs leading to dose reductions: fatigue and hypoalbuminemia, which led to dose reductions in azacitidine from 100 to 75 mg and epacadostat from 100 to 50 mg BID, and decreased appetite and hypersomnia, which contributed to the azacitidine dose reduction. Among the 24 (34.3%) patients who experienced a TEAE leading to dose interruption, nausea (*n* = 3) and fatigue (*n* = 3) were the only events reported in >2 patients. Six (8.6%) patients, all of whom received azacitidine 100 mg and epacadostat 100 mg BID, reported a total of 10 TEAEs leading to the discontinuation of any study drug: disease progression (*n* = 2), abdominal pain (*n* = 1), brain injury (*n* = 1), brain edema (*n* = 1), constipation (*n* = 1), malignant neoplasm progression (*n* = 1), pneumonitis (*n* = 1), proctalgia (*n* = 1), and respiratory failure (*n* = 1). Immune-related TEAEs were reported in four (5.7%) patients (pneumonitis: *n* = 2; hyperthyroidism: *n* = 1; hypothyroidism: *n* = 1); these patients received azacitidine 100 mg and epacadostat 100 mg BID.

### Changes in T-cell infiltration

**Fig. 1:**
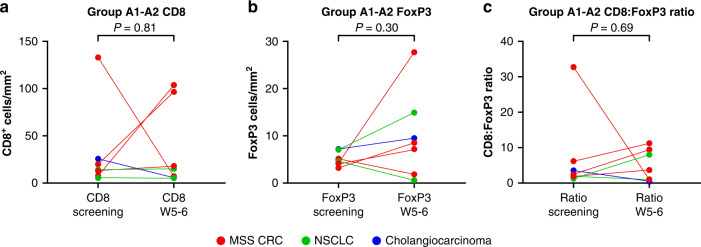
Paired biopsies of tumor-infiltrating lymphocytes in patients administered concurrent treatment with azacitidine, epacadostat, and pembrolizumab (Groups A-1 and A-2). **a** CD8^+^ T-cell density. **b** FoxP3^+^ T-cell density. **c** Ratio of CD8^+^:FoxP3^+^ T cells. CD cluster of differentiation, MSS CRC microsatellite stable colorectal cancer, NSCLC non-small cell lung cancer, W week.

**Fig. 2:**
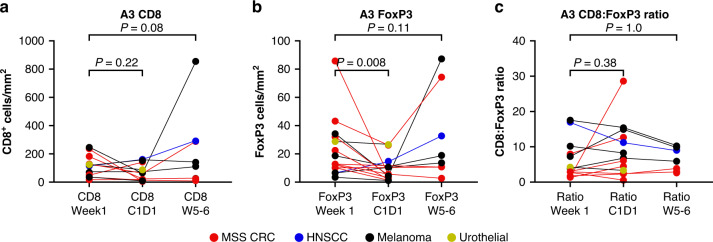
Changes in the intratumoral density of CD8^+^ and FoxP3^+^ T cells relative to the pre-treatment biopsy in patients who received a 7-day run-in of azacitidine followed by the addition of epacadostat and pembrolizumab (Group A-3). **a** CD8^+^ T-cell density. **b** FoxP3^+^ T-cell density. **c** Ratio of CD8^+^:FoxP3^+^ T cells. C cycle, CD cluster of differentiation, D day, HNSCC head and neck squamous cell carcinoma, MSS CRC microsatellite stable colorectal cancer, W week.

**Fig. 3:**
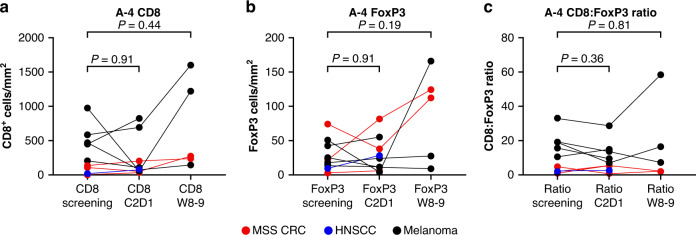
Changes in the intratumoral density of CD8^+^ and FoxP3^+^ T cells relative to the pre-treatment biopsy in patients who received epacadostat and pembrolizumab for one cycle followed by the addition of azacitidine (Group A-4). **a** CD8^+^ T-cell density. **b** FoxP3^+^ T-cell density. **c** Ratio of CD8^+^:FoxP3^+^ T cells. C cycle, CD cluster of differentiation, D day, HNSCC head and neck squamous cell carcinoma, MSS CRC microsatellite stable colorectal cancer, W week.

Evaluable samples (defined as tumor content ≥10%) for paired pre- and on-treatment biopsies were available from seven patients who received concurrent administration of azacitidine, epacadostat, and pembrolizumab (dose escalation: *n* = 3; Group A-1: *n* = 2; Group A-2: *n* = 2). No consistent changes between pre-treatment and on-treatment biopsies were observed for the numbers of intratumoral CD8^+^ or FoxP3^+^ T cells (Fig. 1a, b) or for the ratio of CD8^+^:FoxP3^+^ T cells (Fig. 1c). Fourteen patients who received run-in azacitidine followed by the addition of epacadostat and pembrolizumab (Group A-3) had an evaluable baseline biopsy and ≥1 on-treatment biopsy, with all three biopsies evaluable in seven patients. The azacitidine run-in period resulted in a decrease in the numbers of TILs. There was a trend for a reduction in CD8^+^ T-cell density (*P* = 0.22), whereas the reduction in FoxP3^+^ T-cell density was significant (*P* < 0.05; Fig. 2). Nine patients who received epacadostat and pembrolizumab for one cycle prior to the addition of azacitidine (Group A-4) had evaluable biopsies. No consistent changes in the density of CD8^+^ or FoxP3^+^ T cells or the ratio of CD8^+^:FoxP3^+^ T cells were seen among the paired biopsies (Fig. 3). Representative multiplex immunohistochemistry of tumor samples from a patient with CRC assigned to Group A-4 is shown in additional file 3 (Supplementary Fig. S3).

## Discussion

ECHO-206 represents the largest prospective study of combination treatment with an epigenetic modulator (azacitidine) and immunotherapy (epacadostat plus pembrolizumab) performed to date. Although this study established that azacitidine 100 mg could be safely combined with epacadostat and pembrolizumab, this regimen (with all agents administered concurrently or with a lead-in) was not associated with substantial clinical response in patients with immunotherapy-resistant solid tumors (predominately MSS CRC) or solid tumors that progressed following immunotherapy (predominately melanoma). Whether use of azacitidine could enhance immunotherapy response in the treatment naïve setting is unclear. Our findings contrast with preliminary results from the Phase I/II ENCORE-601 study, which evaluated combination treatment with the DNMT inhibitor entinostat and pembrolizumab in patients with anti-PD-1–refractory advanced tumors [40]. Of the 53 patients with melanoma enrolled in ENCORE-601, 10 exhibited a response (CR: *n* = 1; PR: *n* = 9), corresponding to an ORR of 19%. A Phase I study of entinostat and nivolumab with or without ipilimumab in immunotherapy-naïve advanced solid tumors (ETCTN-9844) reported an ORR of 16% (4/25 evaluable) that included one CR in a patient with triple-negative breast cancer [41]. A 2-week run-in period with entinostat monotherapy did not demonstrate significant changes in the overall CD8/FoxP3 ratio. Upon initiation of immune checkpoint inhibition therapy, an expected increase in the intratumoral CD8/FoxP3 ratio was observed, but no correlation was observed between responses and changes to TILs. Future studies may benefit from additional detailed analyses on the functional status of infiltrating T-cell subsets, because their absolute number may not fully explain any treatment effect or lack thereof. This observation also highlights the general unmet need to identify biomarkers of on-treatment response. Because of the limited sample availability, additional analyses were not possible in the current study.

The strategy of targeting the Trp–Kyn–AhR pathway through IDO1 inhibition experienced a setback following the negative results of the Phase III KEYNOTE-252/ECHO-301 study [29]. However, translational analyses undertaken during the Phase I/IIa CA017-003 study suggest that it may be possible to use an RNA-based composite biomarker to identify patients with tumors that may benefit from the addition of IDO1 inhibition to immunotherapy [42]. In this study, RNA sequencing of serum and tumor samples from patients receiving a combination of the IDO1 inhibitor linrodostat mesylate and nivolumab revealed that the composite of T-cell–inflamed and tryptophan 2,3-dioxygenase 2 (TDO2) gene expression signatures correlated with treatment response. As it is known that TDO2 regulates the Trp–Kyn–AhR pathway in a similar way to IDO1, this finding would support continued pursuit of this pathway as a therapeutic target in cancer. We did not evaluate gene expression changes in the current study and are therefore unable to corroborate the previous finding.

Although preclinical findings strongly suggest an ability of epigenetic modulators to facilitate an influx of T cells into the tumor microenvironment [43], we did not observe an increase in intratumoral CD8^+^ T cells following treatment with azacitidine plus epacadostat and pembrolizumab, irrespective of treatment sequencing. However, a significant reduction in the density of FoxP3^+^ regulatory T cells was measured, but only after the azacitidine monotherapy run-in period. It is possible that additional cycles of azacitidine may have further reduced intratumoral FoxP3 cells, but delaying combination treatment in this patient population was not considered appropriate. This observation contrasts with results from ETCN-9844 where a 2-week entinostat lead-in did not appear to have a similar effect. Future studies will be required to determine if the mechanisms of epigenetic modulation explain this difference and which tumor types are most affected. We cannot discount the possibility that azacitidine (or other epigenetic modifiers) has a direct effect on immune cells. T-cell development and function are driven in part by methylation [44–46], and should this activity have a negative effect on inflammatory processes (eg, IFN-γ induction), sequential therapy may be more effective than combinations. Of note, a Phase II study (NCT01928576) evaluating treatment with the PD-1 inhibitor nivolumab alone and in combination with azacitidine plus entinostat in patients with NSCLC is underway.

With the doses of pembrolizumab and azacitidine used in this study, we believe pharmacodynamic changes and/or any clinical benefit with combination treatment would have been observed. However, dosing of epacadostat may have been insufficient. A longitudinal analysis of plasma samples acquired from participants across multiple clinical studies revealed that epacadostat doses <600 mg BID were not sufficient to reduce plasma kynurenine levels when combined with PD-1 inhibition [47]. IDO1 expression is known to be induced by IFN-y [48]. If epigenetic modifiers result in increased IFN-y expression as expected, this could further contribute to expression of IDO1 at levels that cannot be mitigated by IDO1 inhibitors. To maximize blockade of IDO1 activity in the context of anti-PD-1 treatment, doses of epacadostat higher than those tested in earlier clinical trials and without epigenetic modifiers may be informative. Studies of higher doses of epacadostat are ongoing, with additional proof-of-concept clinical trials planned. In addition, dual IDO/TDO inhibitors, recombinant kynurenine-degrading enzymes, and aryl hydrocarbon receptor antagonists are being evaluated in early-stage clinical trials [10]. Thus, the targeting of the Trp–Kyn–AhR pathway remains under active clinical investigation.

It is apparent that many questions remain regarding the potential of epigenetic modifiers in enhancing the clinical activity of immunotherapy. Other epigenetic modifiers of clinical interest include agents targeting protein arginine methyltransferase 5, SET domain bifurcated, lysine demethylase 1, and cyclin-dependent kinase 9. These agents have all been demonstrated in the preclinical setting to induce viral mimicry responses, regulate type I and II IFN responses, and/or enhance the antitumor activity of PD-(L)1 inhibitors [49–52]. Further studies will be needed to understand which epigenetic modifiers to employ, the optimal sequencing (and timing) of combination regimens, and dosing strategies, all of which may be dependent on tumor histology.

In summary, we performed the largest prospective study to date combining epigenetic modulation (azacitidine) with IDO1 inhibition (epacadostat) and immune checkpoint blockade (pembrolizumab). We demonstrated that both epigenetic lead-in and concurrent therapy were moderately well-tolerated. However, overall efficacy was limited in our cohort of patients, the majority of whom had either prior experience with immunotherapy or had immunotherapy-refractory tumors. Although the DNMT inhibitor azacitidine suppressed influx of intratumoral regulatory T cells, no increase in the numbers of effector T cells was observed, suggesting that the ability of azacitidine to influence intratumoral CD8^+^ T cell infiltration and/or expansion with immunotherapy-based regimens may be limited.

## Supplementary information

Additional File 1

Additional File 2

Additional File 3

## Data Availability

Access to individual patient-level data is not available for this study.
