# Supplementary material for: Phase I/II sequencing study of azacitidine, epacadostat, and pembrolizumab in advanced solid tumors
Source: Br J Cancer. 2023 Apr 22;128(12):2227–35. doi: 10.1038/s41416-023-02267-1 (PMC10241827; doi:10.1038/s41416-023-02267-1)
Supplement: Supplementary file 1 — Additional File 1 [file 41416_2023_2267_MOESM1_ESM.docx]

**ADDITIONAL FILE 1**

**Supplemental Figure 1** Biopsy sequencing cohorts


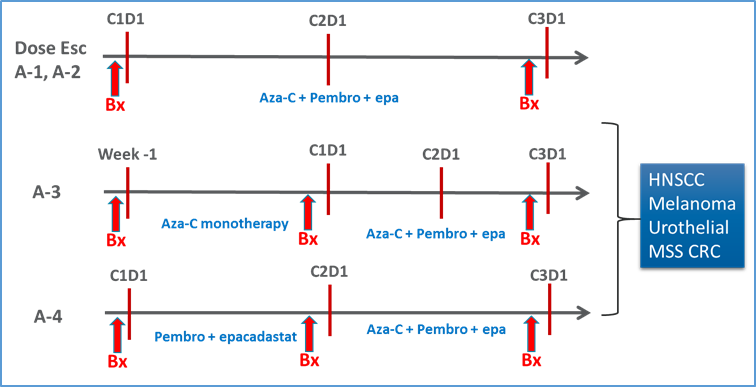


Cycles were 21 days in length.

In groups A-1 and A-2, biopsies were collected at baseline and during week 5 or 6. In group A-3, biopsies were collected at baseline, on C1D1, and during week 5 or 6. In group A-4, biopsies were collected at baseline, C2D1, and during week 8 or 9.

Aza, azacitidine; Bx, biopsy; C, cycle; D, day; epa, epacadostat; esc, escalation; HNSCC, head and neck squamous cell carcinoma; MSS CRC, microsatellite stable colorectal cancer; pembro, pembrolizumab.

**Supplemental Figure 2** Study design for the evaluation of azacitidine plus epacadostat and pembrolizumab


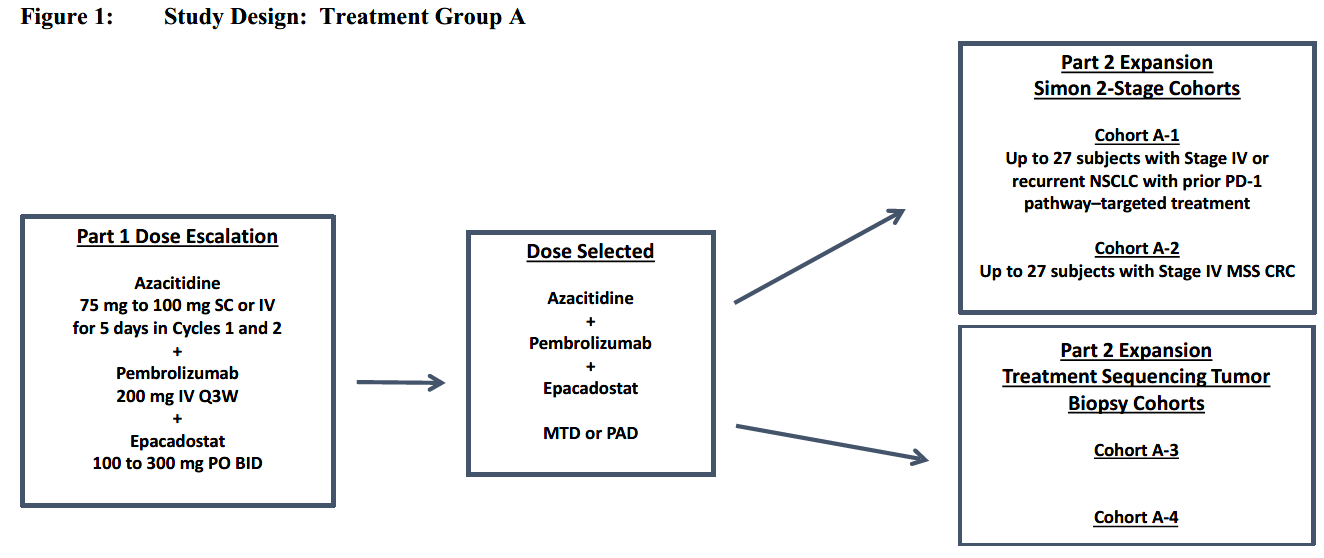


Cohort A-1 and A-2, concurrent administration of azacitidine, epacadostat, pembrolizumab; Cohort A-3, azacitidine run-in followed by addition of epacadostat and pembrolizumab; Cohort A-4, one cycle of epacadostat plus pembrolizumab followed by the addition of azacitidine.

BID, twice daily; IV, intravenous; MSS CRC, microsatellite stable colorectal cancer; MTD, maximum tolerated dose; NSCLC, non-small cell lung cancer; PAD, pharmacologically active dose; PD-1, programmed cell death protein-1; PO, orally; Q3W, every 3 weeks; SC, subcutaneous.
