# Supplementary material for: Phase I/II sequencing study of azacitidine, epacadostat, and pembrolizumab in advanced solid tumors
Source: Br J Cancer. 2023 Apr 22;128(12):2227–35. doi: 10.1038/s41416-023-02267-1 (PMC10241827; doi:10.1038/s41416-023-02267-1)
Supplement: Supplementary file 2 — Additional File 2 [file 41416_2023_2267_MOESM2_ESM.docx]

**ADDITIONAL FILE 2**

**Supplemental Table 1** Definition of a DLT

| **Non-hematologic toxicity**   - Grade 4 (life-threatening) vomiting or diarrhea - Grade 4 electrolyte abnormality - Grade 4 systemic reaction - Any grade ≥3 non-hematologic toxicity, except for the following:   - Transient (≤72 hours) abnormal laboratory values without associated clinically significant signs  or symptoms.   - Nausea, vomiting, and diarrhea adequately controlled with medical therapy within 48 hours.   - Grade 3 rash in the absence of desquamation, with no mucosal involvement, that does not require systemic steroids and that resolves to grade 1 within 14 days.   - An event clearly associated with the underlying disease, disease progression, a concomitant medication, or comorbidity.   - Asymptomatic changes in lipid profiles.   - Singular or non-fasting elevations in blood glucose (ie, blood glucose excursions will be considered toxicities if fasting blood glucose is elevated on two separate occasions).   - Immune-related adverse events of grade 3 or higher that improve to grade ≤1 in <5 days by appropriate care or with corticosteroid therapy. |
| --- |
| **Hematologic toxicity**   - Grade ≥3 thrombocytopenia, with clinically significant bleeding (ie, requires hospitalization, transfusion of blood products, or other urgent medical intervention). - Grade 4 thrombocytopenia of any duration. - Grade 4 neutropenia lasting >3 days. - Grade ≥3 febrile neutropenia (absolute neutrophil count <1.0 × 10^9^/L and fever >101°F/38.5°C). - Grade 4 neutropenia that does not recover to grade ≤2 in ≤3 days after interrupting study drug. - Grade 4 anemia not explained by underlying disease or some other concomitant disorder. |
| **General**   - Patients being unable to receive ≥75% of study drug doses during the DLT observation period because of toxicity, even if the toxicity does not meet the DLT criteria defined above.   Note: Exceptions include the DLT exclusions mentioned above. |
| **Maximum tolerated dose**   - One dose level below that at which at least one-third of patients in a particular cohort have DLTs. DLT will be defined as the occurrence of any of the toxicities in ***this table*** occurring up to and including study day 21. |

DLT, dose-limiting toxicity.
