# Supplementary material for: Phase I/II sequencing study of azacitidine, epacadostat, and pembrolizumab in advanced solid tumors
Source: Br J Cancer. 2023 Apr 22;128(12):2227–35. doi: 10.1038/s41416-023-02267-1 (PMC10241827; doi:10.1038/s41416-023-02267-1)
Supplement: Supplementary file 3 — Additional File 3 [file 41416_2023_2267_MOESM3_ESM.docx]

**ADDITIONAL FILE 3**

**Supplemental Figure 3** Representative multicolor immunohistochemistry of tumor samples from a patient with CRC assigned to group A-4.


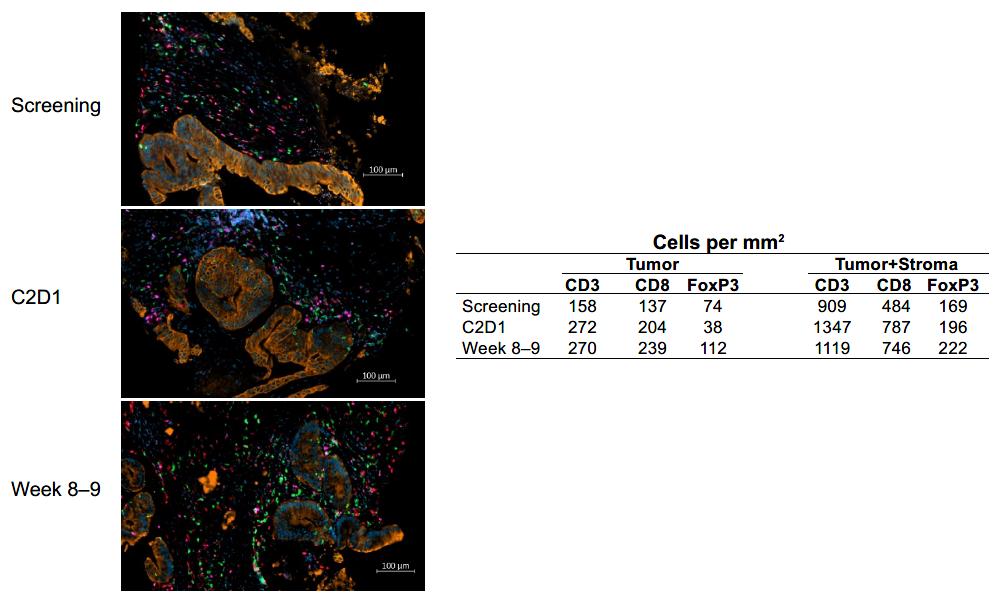


Orange: pan-cytokeratin; red: CD3; green: CD8; magenta: FoxP3; blue: DAPI.

C, cycle; CD, cluster of differentiation; CRC, colorectal cancer; D, day; DAPI, 4′,6-diamidino-2-phenylindole.
